# High accumulation of linezolid and its major metabolite in the serum of patients with hepatic and renal dysfunction is significantly associated with thrombocytopenia and anemia

**DOI:** 10.1128/spectrum.02493-24

**Published:** 2025-05-19

**Authors:** Junqiang Gou, Qian Li, Ning Fan, Chen Zhang, Haiwen Tang, Xiaofeng Wang, Dongfeng Yin

**Affiliations:** 1 Department of pharmacy, General Hospital of Xinjiang Military Command, Urumqi, China; Icahn School of Medicine at Mount Sinai, New York, New York, USA

**Keywords:** linezolid, therapeutic drug monitoring, threshold, adverse reactions, relationship analysis

## Abstract

This study aims to examine the serum levels of linezolid and its metabolites (PNU-142300 and PNU-142586) in patients with varying hepatic and renal function. It seeks to understand how these levels relate to thrombocytopenia and anemia and to identify concentration thresholds that could cause these adverse effects, thereby aiding in personalized drug dosing. This prospective study was conducted from January to December 2023. According to the established inclusion and exclusion criteria, 77 patients with infections treated with linezolid were selected as the research subjects. Venous blood samples were collected every 48 h starting from the first use of linezolid, specifically 30 min before the next dose. Laboratory data were obtained through biochemical analysis and blood routine tests, and blood drug concentration monitoring was carried out based on the pre-established high-performance liquid chromatography (HPLC) method. The exposure levels of linezolid and its metabolites in the serum of patients under different liver and kidney function states were compared, and the relationships between these drug exposure levels and platelet count and hemoglobin concentration were analyzed. Additionally, the receiver operating characteristic (ROC) curve was used to determine the blood drug concentration thresholds of linezolid and its metabolites that led to thrombocytopenia or anemia. Finally, survival analysis was used to evaluate the time differences in the occurrence of adverse reactions, such as thrombocytopenia and anemia, between the liver and kidney function impairment group and the normal group after the use of linezolid. Exposure to linezolid and its metabolites increased with the severity of hepatic and renal impairment. Patients with severe and moderate hepatic and renal impairment had a substantially higher median C_min_ of linezolid and its metabolites 2 and 3 than those with mild hepatic and renal impairment. The platelet count and hemoglobin concentration were significantly associated with linezolid and its metabolite overexposure. The concentration threshold for linezolid and its metabolites 2 and 3 to cause thrombocytopenia and anemia were 7.0, 3.6, and 4.3 mg/L. Patients with hepatic and renal impairment exhibit higher levels of linezolid and its metabolites, potentially leading to adverse effects like thrombocytopenia and anemia. It is recommended to monitor drug levels and develop individualized dosage regimens.

**IMPORTANCE:**

The accumulation of plasma linezolid and its metabolites increased with the degree of liver and kidney injury. High plasma linezolid and its metabolite accumulation is significantly associated with thrombocytopenia and anemia. Linezolid and its metabolite concentration threshold can warn the clinical prevention of hematological adverse reactions. Individual therapy guided by therapeutic drug monitoring (TDM) can improve the efficacy of linezolid and reduce toxic reactions. Patients with severe hepatic and renal dysfunction should actively monitor the blood routine and linezolid concentration and adjust the dosage in time.

## INTRODUCTION

Linezolid, the first synthetic oxazolidinone antimicrobial drug, functions by binding to the 23S rRNA nucleotide site of the bacterial 50S ribosomal subunit. This action impedes the formation of the 70S ribosomal complex by preventing the connection of fMet-RNA to the ribosome, ultimately hindering bacterial protein synthesis and yielding bacteriostatic and bactericidal outcomes (1). In clinical practice, linezolid is predominantly utilized for treating infections instigated by drug-resistant Gram-positive bacteria, exhibiting efficacy against methicillin-resistant *Staphylococcus aureus* (MRSA) and vancomycin-resistant enterococcus (VRE)(2–4).

Linezolid metabolizes into the aminoethoxyacetic acid metabolite PNU-142300 (metabolite 2) via the lactam pathway and the hydroxyethylglycine metabolite PNU-142586 (metabolite 3) via the lactone pathway. Antibacterial activity is absent from both main metabolites (5). Non-renal clearance accounts for approximately 65% of the total clearance of linezolid, with 30% excreted in the urine, 10% as metabolite 2, and 40% as metabolite 3 at steady state. The low renal clearance rate of linezolid (average of 40 mL/min) suggests reabsorption from the tubular nephron (6, 7).

In recent years, the utilization of linezolid in clinical settings has been on the rise as a result of its notable efficacy against multi-drug resistant bacteria. This increased use has resulted in a higher incidence of haematological adverse reactions, the most common of which are thrombocytopenia and anemia, occurring in 30% to 45% of cases (8–10). These adverse reactions were predominantly observed in patients with hepatic and renal impairment, elderly patients, and longer treatment duration (11–13). Linezolid and its metabolites 2 and 3 were found to have significantly higher serum C_min_ in patients with renal impairment than those with normal renal function (14, 15). Similarly, patients with severe hepatic impairment had significantly higher linezolid C_min_ levels compared with those with mild or moderate hepatic impairment. Severe hepatic impairment was found to be significantly associated with linezolid overexposure (16). Patients with moderate to severe hepatic and renal impairment were more likely to experience adverse effects like thrombocytopenia and anemia (17). The aforementioned studies indicate that increased exposure to linezolid and its metabolites in patients with hepatic and renal impairment may play a significant role in the development of hematological adverse effects associated with linezolid. However, the existing studies have not sufficiently elucidated the exposure characteristics of linezolid and its metabolites in patients with different grades of hepatic and renal function.

This study utilized a pre-established high-performance liquid chromatography (HPLC) method to analyze the drug concentrations of linezolid and its metabolites 2 and 3 in patients. The objectives were to examine the cumulative exposures of linezolid and its metabolites in patients with varying levels of hepatic and renal function, analyze the relationship between exposures to linezolid and its metabolites and platelet counts and hemoglobin concentration, and define the concentration thresholds of linezolid and its metabolites for causing thrombocytopenia and anemia in order to provide a realistic basis for the wise use of linezolid.

## MATERIALS AND METHODS

### Study design and data sources

A prospective study was conducted on 363 blood samples from 77 patients with varying levels of hepatic and renal function at a tertiary hospital in Xinjiang from January to December 2023. All patients received linezolid at a standard dose (600 mg every 12 h) administered orally or intravenously. Intravenous blood samples were collected at 48 h intervals starting from the initial dose of linezolid. Blood samples were analyzed for routine blood tests and monitored for serum drug trough concentrations.

### Study subjects

**Fig 1:**
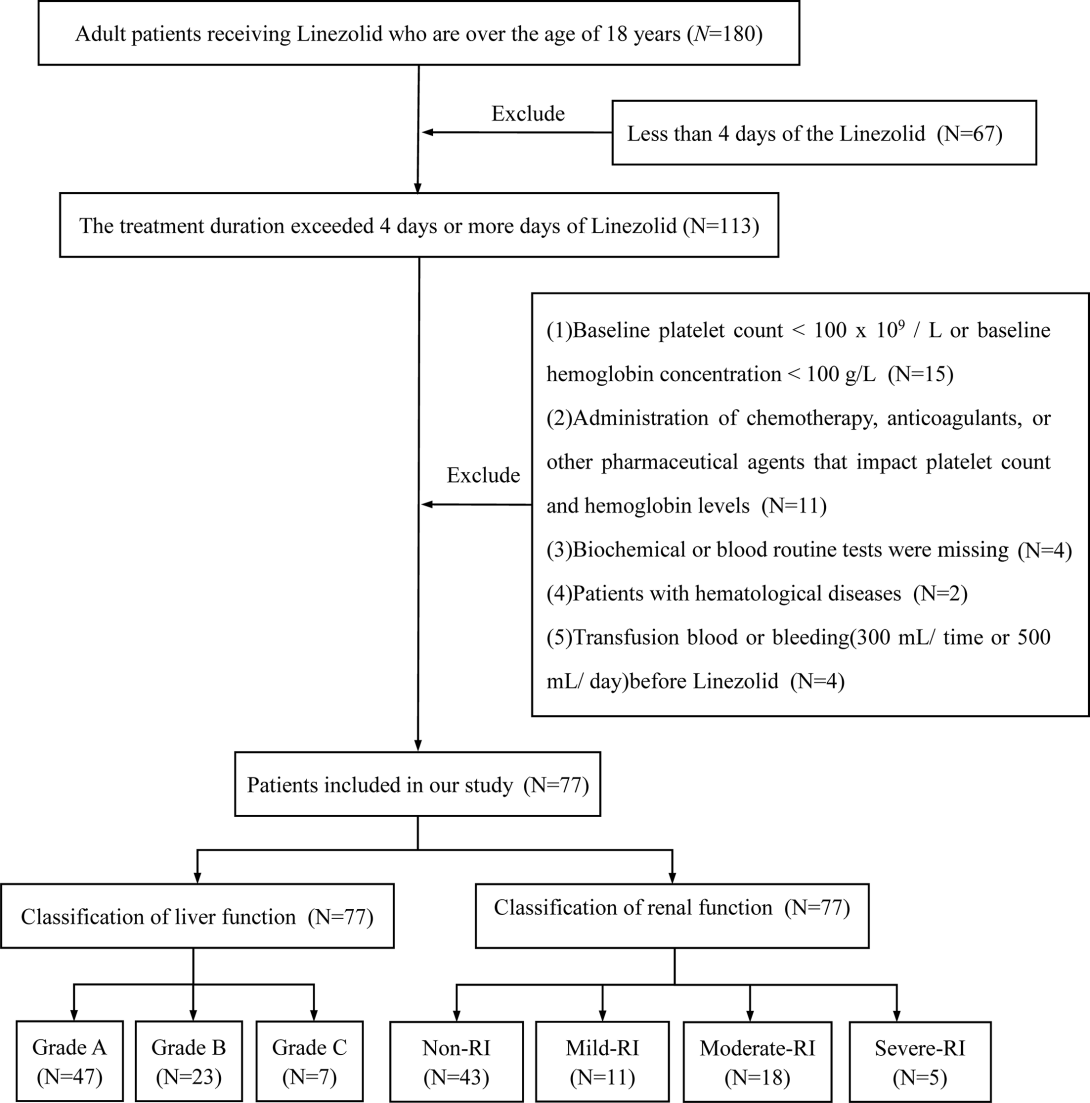
Study grouping and inclusion and exclusion criteria.

Inclusion criteria: (i) age ≥18 years; (ii) use of linezolid to fight infection; exclusion reasons: (i) baseline platelet count <100 × 10^9^/L or baseline hemoglobin concentration <100 g/L; (ii) combined use of antitumor drugs, anticoagulant drugs, or other drugs affecting platelet counts and hemoglobin concentrations; (iii) duration of treatment with linezolid <4 days; (iv) missing baseline blood routine and biochemical indicators; (v) the existence of linezolid allergy; (vi) pregnancy or lactation; (vii) the combination of hematological disorders or rheumatological disorders (8); the occurrence of large bleeding (300 mL/episode or 500 mL/day); (ix) coagulation disorders; (x) history of blood transfusion during drug administration. The disease selection and grouping design of the included studies are shown in Fig. 1, and the case characteristics are shown in Table 1.

**TABLE 1:** Basic clinical characteristics of patients with or without thrombocytopenia and anemia^*d*^

Variables	Thrombocytopenia		*P* value	Anemia		*P* value
	Yes (*n* = 26)	None (*n* = 51)		Yes (*n* = 22)	None (*n* = 55)	
General characteristics						
Sex, n (%)			0.458^*c*^			0.939^*c*^
Male	19 (73.08)	33 (64.71)		15 (68.18)	37 (67.27)	
Female	7 (26.92)	18 (35.30)		7 (31.82)	18 (32.73)	
Age (years)	80.50 (50.50, 88.50)	54.00 (35.00, 72.00)	0.005^*a*^	80.50 (53.00, 88.00)	54.00 (31.00, 75.00)	0.004^*a*^
Duration (days)	11.00 (7.75, 15.00)	10.00 (7.00, 13.00)	0.018^*a*^	12.00 (9.00, 15.00)	10.00 (7.00, 13.00)	0.029^*a*^
Daily dose (mg/kg)	18.50 (15.75, 21.83)	18.50 (16.00, 20.00)	0.336^*a*^	17.10 (14.90, 21.00)	18.50 (16.00, 20.00)	0.483^*a*^
Body weight (kg)	65.00 (55.00, 76.25)	65.00 (60.00, 75.00)	0.336^*a*^	70.005 (7.50, 80.50)	65.00 (60.00, 75.00)	0.483^*a*^
BMI (kg/m^2^)						
Low-weight	18.29 (17.85, 18.30)	18.22 (17.54, 18.29)	0.386^*a*^	18.18 (17.81, 18.24)	18.27 (17.84, 18.30)	0.149^*a*^
Normal	22.61 (21.56, 23.19)	22.44 (21.47, 23.13)	0.678^*a*^	22.68 (21.17, 22.98)	22.46 (21.54, 23.24)	0.910^*a*^
Overweightt	25.92 (24.55, 30.80)	25.99 (24.86, 28.50)	0.818^*a*^	26.99 (24.77, 29.85)	25.95 (24.56, 27.34)	0.439^*a*^
Baseline laboratory parameters, median (IQR)						
Thrombocytopenia	277 (199.25, 363.00)	213 (164.00, 303.00)	0.052^*a*^	201 (127.50, 311.25)	236 (190.00, 316.00)	0.165^*a*^
Anemia	127 (119.75, 137.00)	132 (122.00, 145.00)	0.144^*a*^	125 (118.75, 136.50)	132 (123.00, 144.00)	0.108^*a*^
Serum albumin (g/L)	32.45 ± 0.71	35.99 ± 0.63	0.001^*b*^	31.49 ± 0.84	36.12 ± 0.55	< 0.001^*b*^
ALT (U/L)	23.50 (12.75, 45.00)	24.00 (16.00, 43.00)	0.730^*a*^	31.00 (19.00, 55.50)	22.00 (15.00, 41.00)	0.147^*a*^
AST (U/L)	35.00 (18.00, 51.25)	24.00 (17.00, 43.00)	0.133^*a*^	41.00 (27.75, 58.75)	23.00 (17.00, 41.00)	0.008^*a*^
TBil (μmol/L)	11.30 (8.75, 18.48)	8.70 (7.00, 14.60)	0.032^*a*^	15.15 (10.58, 45.68)	8.70 (7.00, 13.10)	< 0.001^*a*^
BUN (mmol/L)	9.47 (5.49, 14.80)	5.29 (3.78, 8.24)	0.002^*a*^	11.12 (8.38, 15.99)	5.19 (3.78, 8.07)	< 0.001^*a*^
CysC (mg/L)	1.63 (1.16, 2.01)	1.13 (0.92, 1.50)	0.010^*a*^	1.85 (1.38, 2.57)	1.08 (0.90, 1.42)	< 0.001^*a*^
Scr (μmol/L)	83.00 (70.50, 148.25）	68.00 (50.00, 84.00）	0.007^*a*^	119.00 (78.00, 153.00)	67.00 (50.00, 82.00)	< 0.001^*a*^
Ccr (mL/min)	69.36 ± 12.49	105.91 ± 6.77	0.006^*b*^	53.15 ± 6.53	109.74 ± 7.59	< 0.001^*b*^
eGFR (mL/min/1.73 m^2^)	61.15 (42.21, 102.05)	108.78 (67.62, 142.96)	0.002^*a*^	52.50 (43.12, 72.40)	113.69 (81.54, 166.84)	< 0.001^*a*^
Comorbidity, n (%)						
Hypohepatia	18 (69.23)	13 (25.49)	< 0.001^*c*^	18 (81.82)	16 (29.01)	< 0.001^*c*^
Renal impairment	19 (73.08)	15 (29.41)	< 0.001^*c*^	16 (72.73)	15 (27.27)	< 0.001^*c*^
Cardiovascular disease	14 (53.85)	20 (39.22)	0.221^*c*^	12 (54.55)	21 (38.18)	0.190^*c*^
Diabetes mellitus	5 (19.23)	8 (15.69)	0.695^*c*^	5 (22.73)	8 (14.55)	0.387^*c*^
Type of infection, n (%)						
Pneumonia	22 (84.62)	37 (72.55)	0.237^*c*^	19 (86.36)	40 (72.73)	0.202^*c*^
Skin and tissue infections	4 (15.38)	5 (9.80)	0.477^*c*^	3 (13.64)	6 (10.91)	0.709^*c*^
Intracranial infection	1 (3.85)	8 (15.69)	0.259^*c*^	1 (4.55)	8 (14.55)	0.433^*c*^
Bacteremia	1 (3.85)	3 (5.88)	1.000^*c*^	2 (9.09)	2 (3.64)	0.574^*c*^
Microbiological isolate, n (%)						
*Baumanii*	7 (26.92)	15 (29.41)	0.819^*c*^	6 (27.27)	16 (29.09)	0.873^*c*^
*Klebsiella pneumonia*	7 (26.92)	9 (17.65)	0.343^*c*^	6 (27.27)	10 (18.18)	0.371^*c*^
*Pseudomonas aeruginosa*	2 (7.69)	2 (3.92)	0.600^*c*^	2 (9.09)	2 (3.64)	0.574^*c*^
MRSA	5 (19.23)	13 (25.49)	0.539^*c*^	6 (27.27)	12 (21.82)	0.609^*c*^
MSSA	3 (11.54)	6 (11.76)	1.000^*c*^	2 (9.09)	7 (12.73)	1.000^*c*^
Streptococci	5 (19.23)	10 (19.61)	0.968^*c*^	3 (13.64)	12 (21.82)	0.534^*c*^
*Enterococcus faecalis*	2 (7.69)	5 (9.80)	1.000^*c*^	3 (13.64)	4 (7.27)	0.401^*c*^
*Corynebacterium* spp.	2 (7.69)	2 (3.92)	0.600^*c*^	1 (4.55)	3 (5.45)	1.000^*c*^
*Bacillus* spp.	1 (3.85)	2 (3.92)	1.000^*c*^	1 (4.55)	2 (3.64)	1.000^*c*^

^*a*^ Mann–Whitney U test.

^*b*^ Independent sample t test.

^*c*^ Pearson’s chi-squared test.

^*d*^ ALT, alanine aminotransferase; AST, aspartate aminotransferase; TBil, total bilirubin; BUN, blood urea nitrogen; CysC, cystatin C; Scr, serum creatinine; Ccr, creatinine clearance; eGFR, estimated glomerular filtration rate.

#### Sample collection and processing

All patients’ blood samples were obtained on specific days throughout the treatment period, including days 1, 3, 5, 7, 9, 11, 13, and 15, based on the individual treatment course. The time point of blood collection was 30 min before the next administration after the first administration of linezolid in order to collect the serum trough concentration (C_min_). Following collection, the blood samples underwent routine blood tests, and any remaining samples were centrifuged at 15,000 rpm for 10 min. The resulting serum was then separated and stored in a refrigerator at a temperature of −80 °C for analyses.

#### Sample concentration determination

The serum drug concentrations of the patients were determined using a high-performance liquid chromatographic method that had been pre-established (18). The chromatographic method was as follows: Diamonsil C18 column (250 × 4.6 mm, 5 µm); mobile phases: A (acetonitrile), B (0.1 mol/L citric acid–0.2 mol/L disodium hydrogen phosphate buffer solution, pH 3.0); Gradient elution: 0–15 min, A:8% - 50%; 15–25 min, A: 50%; 25–30 min, A: 50%–8%; flow rate: 0.5 mL/min; detection wavelength: 254 nm; column temperature: 30℃; injection volume: 20 µL. The linearity of linezolid was good within the concentration range of 0.5–40 mg/L under the conditions of this chromatography. The lower limit of quantification was 0.5 mg/L, and the limit of detection was 0.1 mg/L. The linearity of PNU-142300 (2) and PNU-142586 (3) was good in the concentration range of 0.5–20 mg/L; the lower limit of quantification was 0.5 mg/L and the limit of detection was 0.2 mg/L.

#### Definition of hepatic and renal impairment

Patients were stratified based on their hepatic function into groups of mild hepatic impairment (grade A: 5–6 points), moderate hepatic impairment (grade B: 7–9 points), and severe hepatic impairment (grade C: 10–15 points) using the Child–Turcotte–Pugh (CTP) estimated score and grading criteria (19). Additionally, patients were categorized according to their glomerular filtration rate (eGFR) adjusted for standard body surface area into groups of normal renal function (eGFR ≥90 mL/min/1.73 m^2^), mild renal impairment (eGFR: 60–89 mL/min/1.73 m^2^), moderate renal impairment (eGFR: 30–59 mL/min/1.73 m^2^), and severe renal impairment (eGFR <30 mL/min/1.73 m^2^)(20–22).

#### Toxicity analysis of linezolid

Toxicity was defined as follows: (i) thrombocytopenia was defined as a baseline platelet count >100 × 10^9^ /L and a decrease of at least 25% from baseline or a final platelet count <100 × 10^9^ /L after linezolid treatment (17, 23). (ii) Anemia was defined as a baseline hemoglobin concentration surpassing >120 g/L and a reduction of at least 25% from baseline or a final hemoglobin concentration below <100 g/L after linezolid treatment (24).

#### Statistical analyses

SPSS 26.0 statistical software was used for data analysis. To evaluate the normal distribution of the patients‘ basic data information, the Kolmogorov–Smirnov test was initially conducted. Variables that exhibited normal distribution were presented as mean ± standard deviation and analyzed using the paired samples *t*-test. Non-normally distributed measures were represented as M (Q_1_, Q_3_) and compared using the Mann–Whitney U test. Categorical data were expressed as cases (%) and analyzed using the Pearson’s χ test. When the number of grids with theoretical frequency below 5 exceeded 20%, Fisher’s exact test was used. The serum drug concentrations of patients were quantified as M (Q_1_, Q_3_), compared by Mann–Whitney U test between two independent samples, and between three groups and more by Kruskal–Wallis test; *P* < 0.05, which was considered statistically significant. Survival analysis was used to compare the duration of thrombocytopenia and anemia in patients with normal and abnormal hepatic and renal function, and the log-rank test was used to compare whether the difference between the two groups was statistically significant.

## RESULTS

### Comparison of clinical characteristics of patients with or without thrombocytopenia and anemia

According to the specified criteria for inclusion and exclusion in Fig. 1, an analysis was conducted on the fundamental clinical data of 77 patients who satisfied the criteria for the study, with the outcomes presented in Table 1. Of the 77 patients, 26 (33.77%) exhibited thrombocytopenia. There were significant differences in age, treatment duration, serum albumin, total bilirubin (TBil), blood urea nitrogen (BUN), cystatin C (CysC), serum creatinine (Scr), creatinine clearance (Ccr), glomerular filtration rate (eGFR), hepatic and renal impairment between patients with and without thrombocytopenia (*P* < 0.05). In addition, 22 (28.57%) developed anemia. Significant differences were observed in variables, such as age, treatment duration, serum albumin, aspartate transaminase (AST), total bilirubin (TBil), blood urea nitrogen (BUN), cystatin C (CysC), serum creatinine (Scr), creatinine clearance (Ccr), glomerular filtration rate (eGFR), hepatic and renal impairment in patients with and without anemia (*P* < 0.05). Before starting anti-infective therapy with linezolid, 46 patients had a definite diagnosis of Gram-positive infections. *Staphylococcus aureus*, *Baumannii*, and *Klebsiella pneumoniae* were the most frequently isolated microorganisms. In all included patients, the main indication for linezolid was pulmonary infections (75.6%), followed by skin and soft tissue infections (20.8%).

### Cumulative exposure of linezolid and its metabolites in patients with hepatic and renal impairment

**Fig 2:**
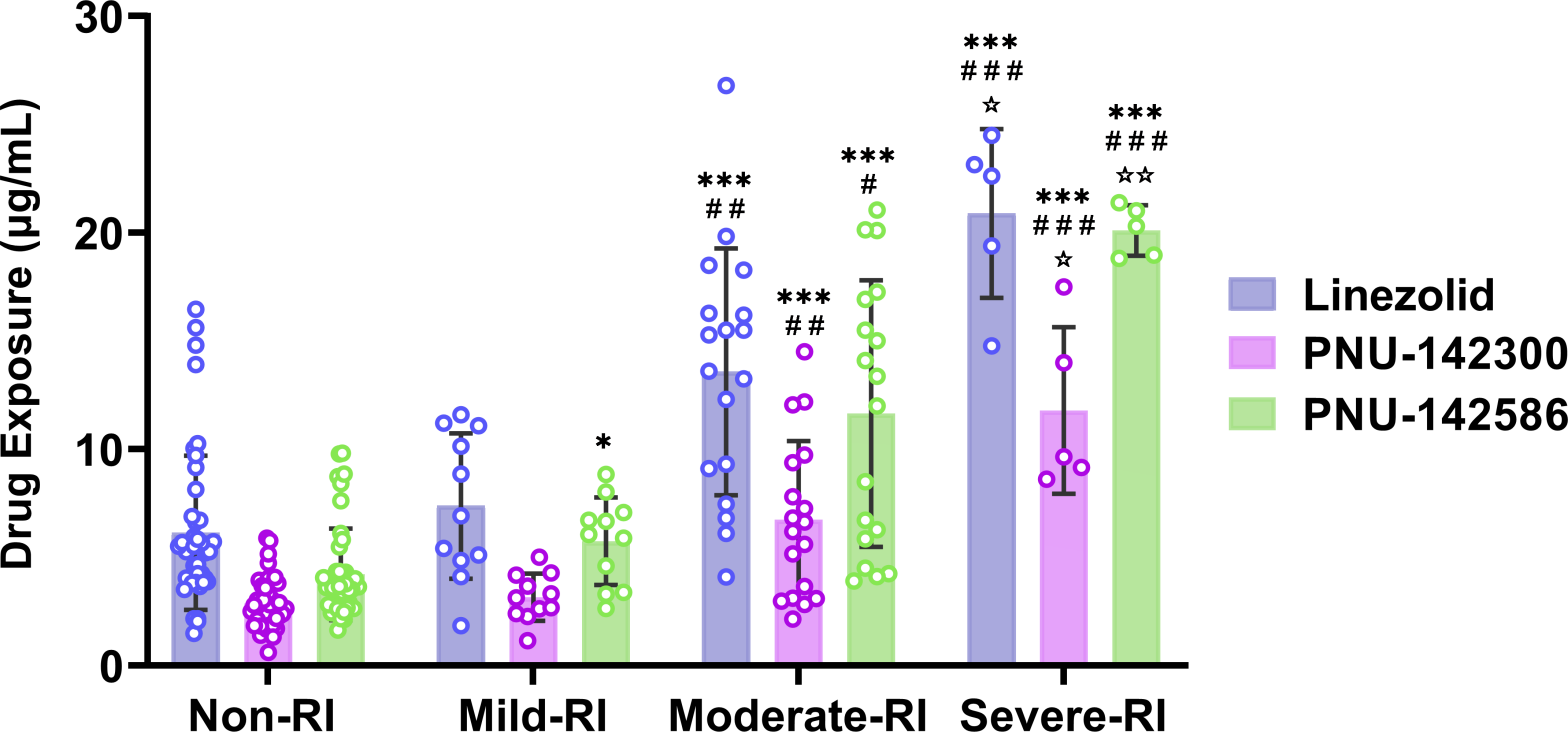
Serum linezolid and its metabolites' cumulative exposure at different renal function levels. Note: Compared with the non-renal impairment group, *P < 0.05, **P < 0.01, ***P < 0.001; Compared with the mild renal dysfunction group, #*P* < 0.05, ##*P* < 0.01; Compared with the moderate renal impairment group, ☆*P* < 0.05, ☆☆*P* < 0.01.

**Fig 3:**
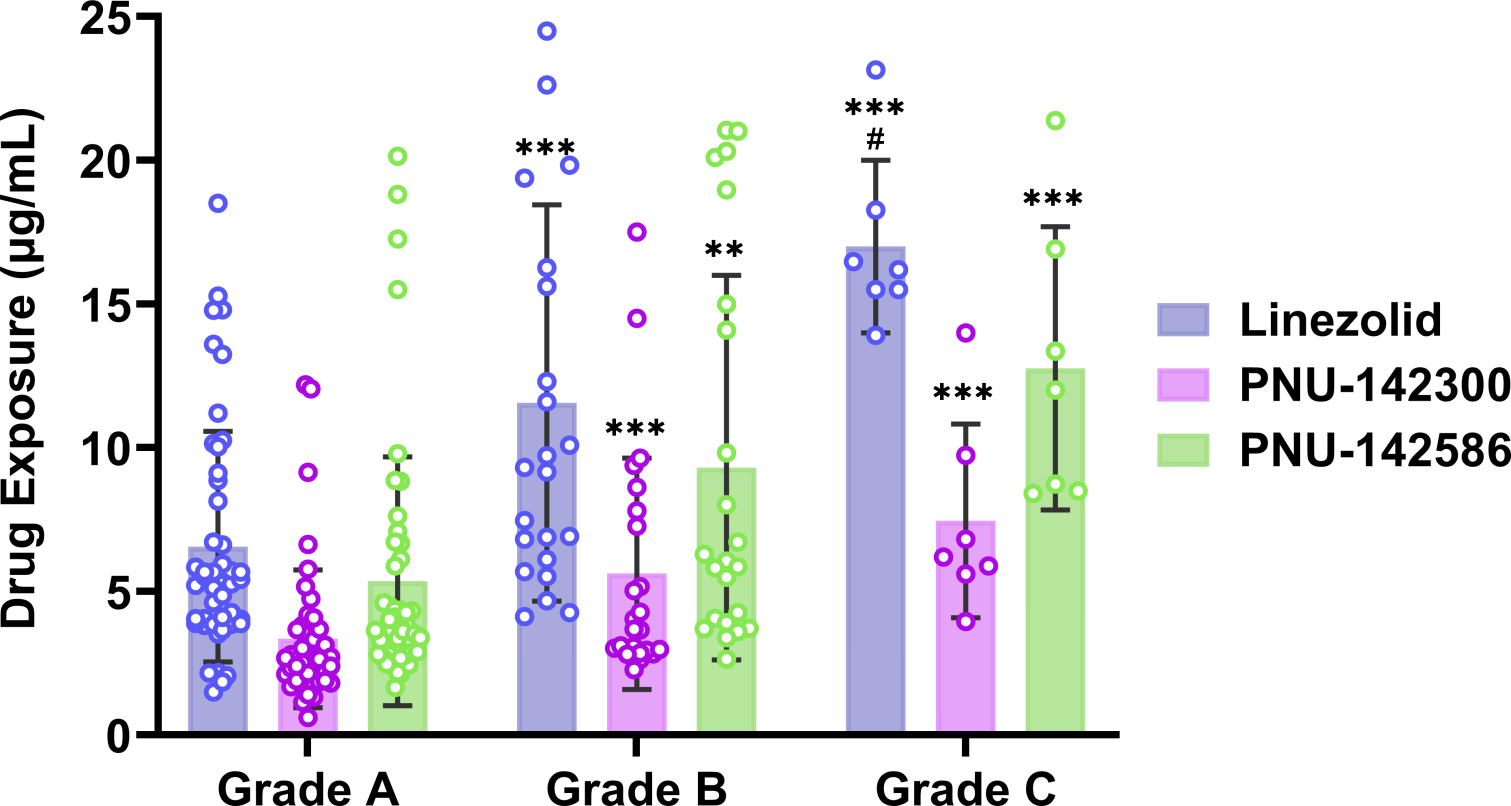
Serum linezolid and its metabolite cumulative exposure at different levels of hepatic function. Note: Compared with the hepatic function grade A, *P < 0.05, **P < 0.01, ***P < 0.001; Compared with grade B hepatic function, #*P* < 0.05, ##*P* < 0.01.

Analysis of cumulative exposure of linezolid and its metabolites in serum of patients with varying levels of hepatic and renal functions is presented in Table 2, Fig. 2 and 3. In comparison to the cohort exhibiting normal renal function, no statistically significant differences were observed in the exposure levels of linezolid and its metabolite 2 within the group characterized by mild renal impairment (*P* > 0.05). Furthermore, the exposure levels of linezolid in this group remained within the effective therapeutic range (C_min_: 2–8 mg/L) (25, 26). Conversely, in the groups with moderate and severe renal insufficiency, significant differences in the exposure levels of linezolid and its metabolites were identified (*P* < 0.001). Specifically, in patients with moderate renal impairment, the serum concentrations of linezolid, metabolite 2, and metabolite 3 were found to be 3.02 times (14.14 mg/L vs 4.68 mg/L), 2.40 times (5.69 mg/L vs 2.37 mg/L), and 3.47 times (11.76 mg/L vs 3.39 mg/L) greater, respectively, than those observed in the normal renal function group. In patients with severe renal impairment, the serum levels of linezolid, metabolite 2, and metabolite 3 were 4.56 times (21.36 mg/L vs 4.68 mg/L), 4.07 times (9.64 mg/L vs 2.37 mg/L), and 5.60 times (18.97 mg/L vs 3.39 mg/L) higher, respectively, compared with the normal renal function cohort.

**TABLE 2:** Exposure of plasma linezolid and its metabolites in patients with different levels of hepatic and kidney function^*a*^

Hepatic and renal function levels		Sample concentration from patients (mg/L)			Thrombocytopenia *N* (%)	Anemia *N* (%)
		Linezolid	PNU, 142300 (2)	PNU, 142586 (3)		
Renal function (*n* = 77)	Normal (*n* = 43)	4.68 (3.65, 7.21)	2.37 (1.84, 3.34)	3.39 (2.68, 4.32)	7 (16.28)	4 (9.30)
	Mild (*n* = 11)	6.92 (4.86, 10.14)	2.69 (2.39, 4.19)	6.06 (3.39, 7.08)	6 (54.55)	3 (27.27)
	Moderate (*n* = 18)	14.14 (8.95, 15.52)	5.69 (3.12, 9.30)	11.76 (5.52, 16.45)	9 (50.00)	12 (66.67)
	Severe (*n* = 5)	21.36 (17.08, 22.62)	9.64 (8.89, 14.83)	18.97 (18.47, 20.46)	4 (80.00)	3 (60.00)
Hepatic function (*n* = 77)	Grade A (*n* = 47)	5.12 (3.65, 8.86)	2.37 (1.84, 3.60)	3.48 (2.68, 6.12)	8 (17.02)	6 (12.77)
	Grade B (*n* = 23)	9.31 (6.11, 15.62)	3.69 (2.92, 7.32)	6.06 (3.91, 14.57)	14 (60.87)	10 (43.48)
	Grade C (*n* = 7)	15.46 (15.30, 16.47)	5.89 (5.02, 9.27)	11.58 (8.41, 16.70)	4 (57.14)	6 (85.71)

^*a*^ Normal renal function: eGFR ≥90 mL/min/1.73 m^2^; mild renal impairment: eGFR 60–89 mL/min/1.73 m^2^; moderate renal impairment: eGFR 30–59 mL/min/1.73 m^2^; severe renal impairment: eGFR <30 mL/min/1.73 m^2^. According to the Child, Turcotte, Pugh score, the hepatic function was divided into Grade A (5–6 points), Grade B (7–9 points), and Grade C (≥10 points).

In comparison to the cohort exhibiting mild renal impairment, the differences in serum exposure levels of linezolid and its metabolites among patients with moderate and severe renal insufficiency were found to be statistically significant (*P* < 0.01). Specifically, the serum concentrations of linezolid, metabolite 2, and metabolite 3 in patients with moderate renal impairment were 2.04 times (14.14 mg/L vs 6.92 mg/L), 2.11 times (5.69 mg/L vs 2.69 mg/L), and 1.94 times (11.76 mg/L vs 6.06 mg/L) higher, respectively, than those observed in the mild renal impairment group. In patients with severe renal impairment, the serum levels of linezolid, metabolite 2, and metabolite 3 were 3.08 times (21.36 mg/L vs 6.92 mg/L), 3.58 times (9.64 mg/L vs 2.69 mg/L), and 3.13 times (18.97 mg/L vs 6.06 mg/L) greater, respectively, than those in the mild renal impairment group. In comparison to the group exhibiting moderate renal impairment, the differences in serum exposure levels of linezolid and its metabolites in patients with severe renal impairment were found to be statistically significant (*P* < 0.05). Specifically, the serum exposure levels of linezolid, metabolite 2, and metabolite 3 in patients with severe renal impairment were 1.51 times (21.36 mg/L vs 14.14 mg/L), 1.69 times (9.64 mg/L vs 5.69 mg/L), and 1.61 times (18.97 mg/L vs 11.76 mg/L) greater than those observed in the moderate renal impairment group, respectively.

In comparison to the mild hepatic impairment group, statistically significant differences were observed in the exposure of linezolid and its metabolites in the moderate and severe hepatic impairment groups (*P* < 0.001). Specifically, serum concentrations of linezolid, metabolite 2, and metabolite 3 were found to be 1.82 times (9.31 mg/L vs 5.12 mg/L), 1.56 times (3.69 mg/L vs 2.37 mg/L), and 1.74 times (6.06 mg/L vs 3.48 mg/L) higher, respectively, in patients with moderate hepatic impairment. In patients with severe hepatic impairment, the serum levels of linezolid, metabolite 2, and metabolite 3 were 3.02 times (15.46 mg/L vs 5.12 mg/L), 2.49 times (5.89 mg/L vs 2.37 mg/L), and 3.33 times (11.58 mg/L vs 3.48 mg/L) greater, respectively, compared with those with mild hepatic impairment. In comparison to the moderate hepatic impairment group, only the exposure to linezolid in patients with severe hepatic impairment exhibited a statistically significant difference (*P* < 0.05). Although no statistically significant differences were observed in the exposure levels of metabolites 2 and 3 (*P* > 0.05), the serum concentrations of both metabolites were nonetheless elevated. This variation in exposure may primarily be attributed to the accumulation of linezolid resulting from the diminished metabolic capacity of the hepatic following the progression of hepatic injury. Specifically, serum concentrations of linezolid, metabolite 2, and metabolite 3 in patients with severe hepatic impairment were 1.66 times (15.46 mg/L vs 9.31 mg/L), 1.60 times (5.89 mg/L vs 3.69 mg/L), and 1.91 times (11.58 mg/L vs 6.06 mg/L), respectively, higher than those observed in patients with moderate hepatic impairment.

Analysis of the incidence of thrombocytopenia and anemia in patients with different hepatic and renal function grades showed that patients with moderate and severe hepatic and renal impairment showed a higher incidence of adverse reactions, which may be caused by high exposure to linezolid and its metabolites.

### Relationship between exposure to linezolid and its metabolites and platelet count and hemoglobin concentration

**Fig 4:**
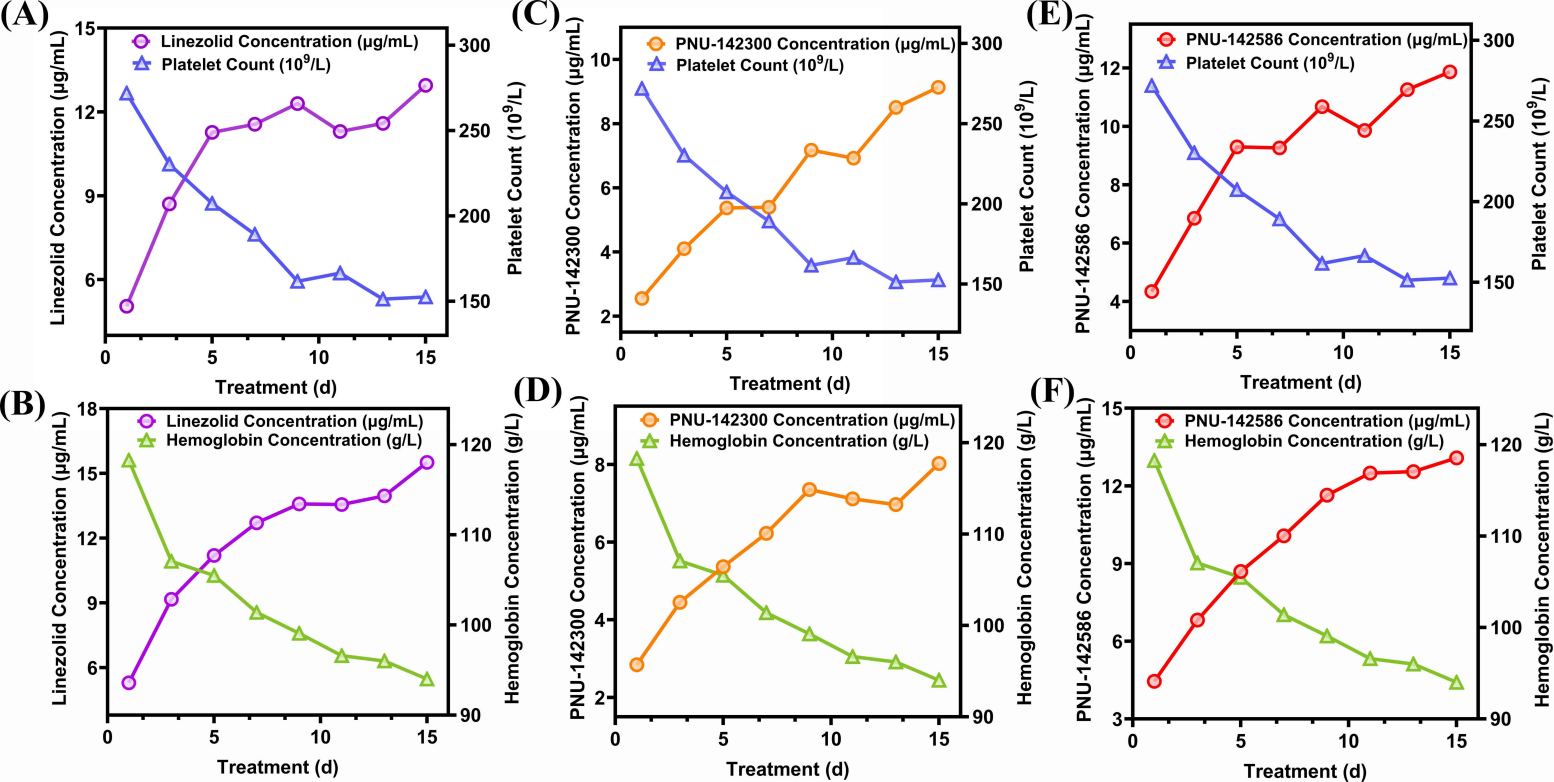
Regression between serum linezolid and its metabolite cumulative exposure and platelet count and hemoglobin concentration. Note: (**A**) Correlation between linezolid and platelet count; (**B**) correlation between linezolid and hemoglobin concentration; (**C**) correlation between PUN-142300 (2) and platelet count; (**D**) correlation between PUN-142300 (2) and hemoglobin concentration; (**E**) correlation between PNU-142586 (3) and platelet count; (F) correlation between PNU-142586 (3) and hemoglobin concentration.

As shown in Fig. 4 and Table 3, the exposure (X) of linezolid and its metabolites 2 and 3 in serum of 26 patients with thrombocytopenia was strongly negatively related to platelet count (Y) (Y = −13.84X +342.1, *r* = 0.914, *P* < 0.01; Y = −17.41X + 300.1, *r* = −0.934, *P* < 0.001; Y = −16.02X + 340.1, *r* = −0.961, *P* < 0.001). The exposure (X) of linezolid and its metabolites 2 and 3 in serum of 22 patients with anemia was strongly negatively related to hemoglobin concentration (Z) (Z = −2.342X + 130.2, *r* = −0.979, *P* < 0.001; Z = −4.568X + 129.5, *r* = −0.978, *P* < 0.001; Z = −2.404X + 126.4, *r* = −0.9528, *P* < 0.001). These results indicated that the occurrence of thrombocytopenia and anemia was significantly related to the patient’s serum drug exposure, which will further promote the rational use of linezolid in clinical practice.

**TABLE 3:** Regression analysis of plasma linezolid and its metabolite exposure with platelet count and hemoglobin concentration in patients with thrombocytopenia and anemia

Sample concentration (mg/L)	Platelet count (*n* = 26)			Hemoglobin concentration (*n* = 22)		
	Formula	R	*P* value	Formula	R	*P* value
Linezolid	Y = 13.84X + 342.1	0.914	< 0.01	Z = 2.342X + 130.2	0.979	<0.001
PNU-142300	Y = 17.41X + 300.1	0.934	< 0.001	Z = 4.568X + 129.5	0.978	<0.001
PNU-142586	Y = 16.02X + 340.1	0.961	< 0.001	Z = 2.404X + 126.4	0.9528	<0.001

### Concentration thresholds for linezolid and its metabolites 2 and 3 to cause thrombocytopenia and anemia

**Fig 5:**
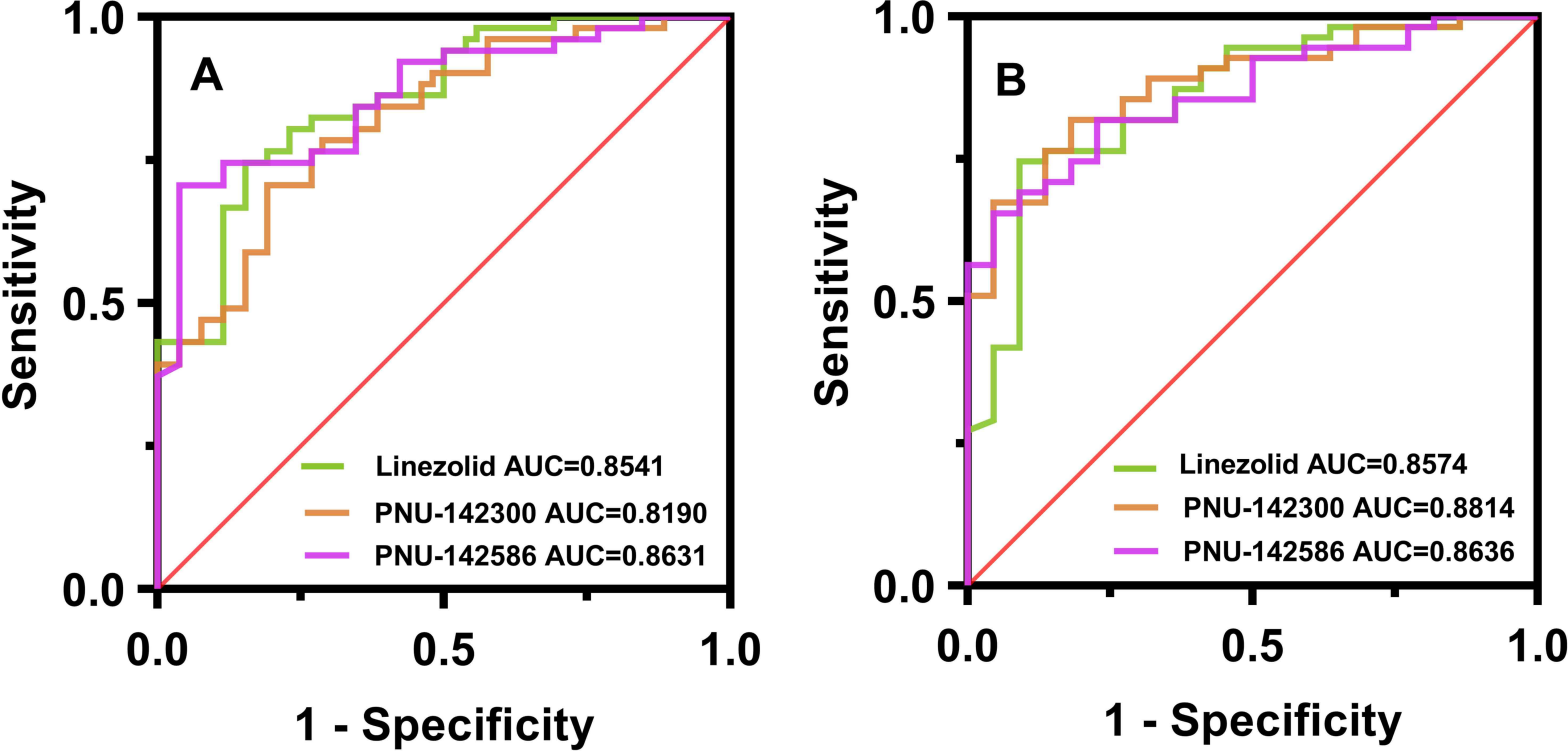
(**A**) Linezolid-related thrombocytopenia ROC curve; (**B**) linezolid-related anemia ROC curve.

To determine whether thrombocytopenia and anemia as the dependent variables, the receiver-operating characteristic curve (ROC curve) of patients with serum linezolid and its metabolites exposure ws analyzed, and the result is shown in Fig. 5 and Table 4. The incidence of thrombocytopenia and anemia can be accurately predicted by linezolid and its metabolites. The area under the receiver-operating characteristic curve (AUC) was greater than 0.85. In addition, the optimal diagnostic concentrations of linezolid and its metabolites 2 and 3 were obtained when the Youden index was maximum. Among which, the optimal diagnostic concentrations of linezolid for predicting thrombocytopenia and anemia were 6.905 and 7.195 mg/L, respectively. The optimal diagnostic concentration of metabolite 2 for predicting thrombocytopenia and anemia was 3.230 and 3.885 mg/L, respectively, and metabolite 3 for predicting thrombocytopenia and anemia was 4.295 mg/L.

**TABLE 4:** Concentration thresholds for linezolid and its metabolites 2 and 3 associated with thrombocytopenia and anemia

Classify	Target	Sensitivity	Specificity	Critical value	AUC	95% CI	*P* value
Thrombocytopenia	Linezolid	84.62%	74.51%	6.905	0.854	0.768–0.941	<0.001
	PNU-142300	80.77%	70.59%	3.230	0.819	0.724–0.914	<0.001
	PNU-142586	96.15%	70.59%	4.295	0.863	0.782–0.944	<0.001
Anemia	Linezolid	90.91%	74.55%	7.195	0.857	0.765–0.950	<0.001
	PNU-142300	81.82%	81.82%	3.885	0.881	0.806–0.957	<0.001
	PNU-142586	95.45%	65.45%	4.295	0.864	0.784–0.943	<0.001

Although no previous reports have investigated the threshold serum trough concentrations of linezolid metabolites 2 and 3, studies of linezolid-induced trough concentrations of thrombocytopenia and anemia have shown an increased risk of adverse effects when the cumulative trough concentration (C_min_) of linezolid reaches 7–8 mg/L (27, 28). Therefore, the concentration thresholds of linezolid and its metabolites 2 and 3 to cause thrombocytopenia and anemia could be determined as 7.0 mg/L (AUC = 0.8558, 95% CI: 0.7664–0.9451, *P* < 0.001), 3.6 mg/L (AUC = 0.8502, 95% CI 0.7647–0.9357, *P* < 0.001), and 4.3 mg/L (AUC = 0.8634, 95% CI 0.7831–0.9737, *P* < 0.001). When the trough concentrations of linezolid and its metabolites 2 and 3 reached this threshold, the increased risk of thrombocytopenia and anemia suggests that the dose of linezolid should be adjusted according to the patient’s hepatic and renal function to ensure the safety of clinical application of linezolid.

### Toxicity analyses

**Fig 6:**
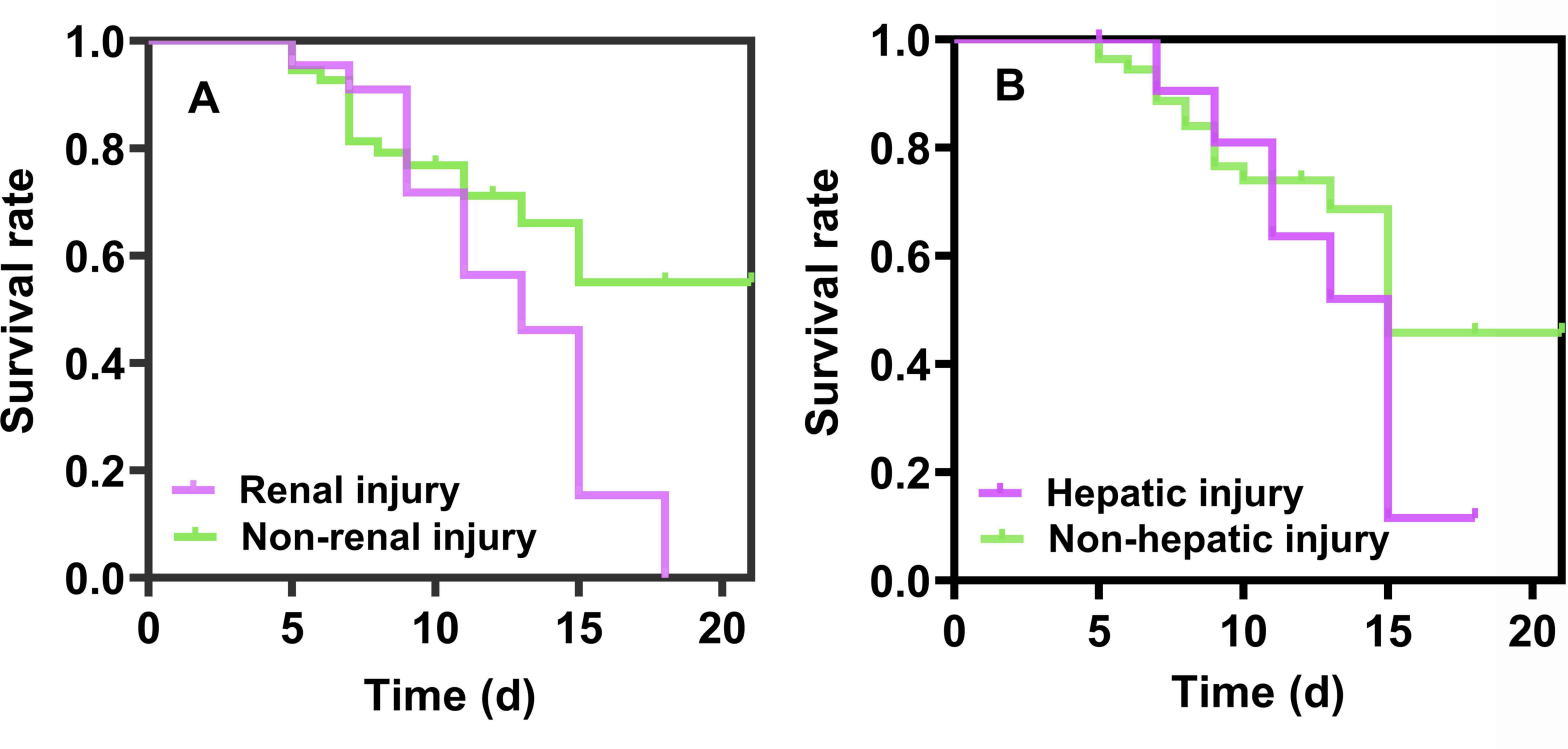
Toxicity analysis of linezolid-associated thrombocytopenia and anemia.

According to the renal function evaluation standard eGFR (A) and hepatic function evaluation index Child–Turcotte–Pugh (CTP) estimated score (B), the toxicity time of thrombocytopenia and anemia in patients with normal and abnormal hepatic and renal function was investigated. The results in Fig. 6 show that patients with hepatic and renal impairment had higher hematologic toxicity than those with normal hepatic and renal function within the same treatment time, and the median toxicity time of patients with hepatic and renal impairment was about 12 days. In patients with normal hepatic and renal function, the median toxicity time is significantly greater than 15 days, and no longer increases with the extension of treatment toxicity, with a statistically significant difference between the two groups (*P* < 0.001).

## DISCUSSION

Linezolid, a potent antibacterial agent, has demonstrated a notable increase in clinical utilization while also presenting a high incidence of adverse reactions. Numerous studies have developed risk prediction models based on clinical data to assess the likelihood of adverse reactions, such as thrombocytopenia and anemia, associated with linezolid use (8, 24, 29). However, according to the risk prediction model, the risk of adverse reactions can only be predicted, and the exposure amount of serum linezolid and its metabolites cannot be obtained. Subsequent dose adjustment still needs to be carried out according to the results of Therapeutic Drug Monitoring (TDM). This study investigates the serum exposure of linezolid and its metabolites in patients with varying hepatic and renal function through TDM. The aim is to explore the association between the exposure levels of linezolid and its metabolites and adverse reactions, particularly thrombocytopenia and anemia. Additionally, the study defined the concentration threshold at which linezolid and its metabolites induce thrombocytopenia and anemia.

Souza et al. (14) found that adverse reactions, such as thrombocytopenia and anemia, caused by linezolid were related to the cumulative exposure of serum linezolid, but there was no significant relationship between linezolid and its major metabolites. This study assessed the serum concentrations of linezolid and its metabolites in patients with varying degrees of hepatic and renal function. The findings indicated that there was no significant difference in serum concentrations of linezolid and its metabolites between patients with normal renal function and those with mild renal impairment (*P* > 0.05). However, a statistically significant difference was observed between patients with moderate to severe hepatic and renal impairment and those with mild hepatic and renal impairment (*P* < 0.01).

Sakurii et al. (30) found that through a population pharmacokinetic model of linezolid and its major metabolites in adult patients, the predicted serum concentrations of linezolid and its two major metabolites accumulate exponentially with the decline in renal function. This finding is consistent with our study. Our results show that the cumulative exposure of linezolid and its metabolites increases in relationship with the severity of hepatic and renal impairment. Patients with moderate and severe hepatic and renal impairment may exceed the upper therapeutic threshold of 8 mg/L for linezolid exposure, while the exposure levels of linezolid and its metabolites in patients with severe hepatic and renal impairment are similar. These results suggest that hepatic and renal functions significantly influence the metabolism and elimination of linezolid and its metabolites from the body. Therefore, for patients with moderate to severe hepatic and renal impairment, particularly those requiring prolonged medication regimens, vigilant monitoring of serum drug concentrations is recommended during linezolid therapy. Adjusting the dosage in a timely manner based on monitoring outcomes can promote the safe and rational utilization of linezolid in clinical settings.

The study found a strong relationship between serum levels of linezolid and its metabolites 2 and 3 and platelet count and hemoglobin concentration in patients with thrombocytopenia and anemia. This suggests that these hematological adverse reactions may be due to cumulative exposure to the drug and its metabolites.

Analysis of linezolid-induced thrombocytopenia and anemia showed that the risk of adverse effects increased when the C_min_ of linezolid was higher than 7–8 mg/L (31), a concentration threshold that has been used as the reference concentration range for individualized treatment of linezolid (32–35). Some studies have found that when the C_min_ of linezolid is maintained at 2–8 mg/L by adjusting the dose, thrombocytopenia and anemia do not occur, and the clinical treatment is effective (34). At present, there are relatively few studies on the C_min_ of the main metabolites of linezolid at home and abroad, and there is no report on the C_min_ threshold of the main metabolites of linezolid to cause thrombocytopenia and anemia. This study identified the trough concentration threshold of metabolites 2 and 3 that can cause thrombocytopenia and anemia in patients. When the C_min_ of linezolid and its metabolites 2 and 3, respectively, is 7.0, 3.6, and 4.3 mg/L, patients have an increased risk for thrombocytopenia and anemia.

This study is limited by a relatively small sample size and was conducted within a single medical institution, which may constrain the representativeness of the sample as patient selection could be influenced by geographical location, accessibility to medical services, and patient preferences. Consequently, future research should aim to enhance the accuracy of determining the concentration thresholds of linezolid metabolites 2 and 3 that cause adverse reactions by engaging in ongoing data collection and validation across a broader, multicenter patient population. Furthermore, the linezolid concentration thresholds established in this study provide a significant reference for clinical drug monitoring, aiding in the guidance of drug dosage adjustment and surveillance in clinical practice. This finding holds potential significance for the optimization of linezolid’s clinical application.
